# Photocatalytic and Antibacterial Properties of a 3D Flower-Like TiO_2_ Nanostructure Photocatalyst

**DOI:** 10.1155/2021/3839235

**Published:** 2021-09-27

**Authors:** Yunping Zhang, Xi Liu, Mahani Yusoff, Mohd Hasmizam Razali

**Affiliations:** ^1^Department of Central Sterile Supply, The Second Affiliated Hospital of Xi'an Jiaotong University, Xi'an 710004, China; ^2^Department of Nursing, The Third Affiliated Hospital of Air Force Medical University, Xi'an 710032, China; ^3^Faculty of Bioengineering and Technology, Universiti Malaysia Kelantan, 17600 Jeli, Kelantan, Malaysia; ^4^Advanced Nanomaterials Research Group, Faculty of Science and Marine Environment, Universiti Malaysia Terengganu, 21030 Kuala Nerus, Terengganu, Malaysia; ^5^Faculty of Science and Marine Environment, Universiti Malaysia Terengganu, 21030 Kuala Nerus, Terengganu, Malaysia

## Abstract

Flower-like titanium dioxide (TiO_2_) nanostructures are successfully synthesized using a hybrid sol-gel and a simple hydrothermal method. The sample was characterized using various techniques to study their physicochemical properties and was tested as a photocatalyst for methyl orange degradation and as an antibacterial material. Raman spectrum and X-ray diffraction (XRD) pattern show that the phase structure of the synthesized TiO_2_ is anatase with 80-100 nm in diameter and 150–200 nm in length of flower-like nanostructures as proved by field emission scanning electron microscope (FESEM). The energy-dispersive X-ray spectroscopy (EDS) analysis of flower-like anatase TiO_2_ nanostructure found that only titanium and oxygen elements are present in the sample. The anatase phase was confirmed further by a high-resolution transmission electron microscope (HRTEM) and selected area electron diffraction (SAED) pattern analysis. The Brunauer-Emmett-Teller (BET) result shows that the sample had a large surface area (108.24 m^2^/g) and large band gap energy (3.26 eV) due to their nanosize. X-ray photoelectron spectroscopy (XPS) analysis revealed the formation of Ti^4+^ and Ti^3+^ species which could prevent the recombination of the photogenerated electron, thus increased the electron transportation and photocatalytic activity of flower-like anatase TiO_2_ nanostructure to degrade the methyl orange (83.03%) in a short time (60 minutes). These properties also support the good performance of flower-like titanium dioxide (TiO_2_) nanostructure as an antibacterial material which is comparable with penicillin which is 13.00 ± 0.02 mm inhibition zone against Staphylococcus aureus.

## 1. Introduction

Generally, a good photocatalyst material must have superior properties like photoactive, able to absorb visible and UV light, biologically and chemically inert, photostable, inexpensive, and nontoxic. Titanium dioxide (TiO_2_) had been reported active and most suitable to be used as a photocatalyst because it is easily available, cheap, and nontoxic [1, 2] and due to its chemical inertness [3]. The surface of TiO_2_ is believed to have high oxidation potential which makes it able to break down many organic substances in photocatalysis process. Photocatalysis is referred to the reaction that uses light to activate a photocatalyst and then increase the speed of chemical reaction by lowering the activation energy for the primary reaction to occur [4]. Photocatalytic reaction in general involved six photocatalytic processes which are (1) charge separation process, (2) surface recombination, (3) surface trapping, (4) surface recombination, (5) interfacial charge transfer, and (6) back reaction [5]. This process occurred when the absorption of light generates an electron-hole pair on the surface of TiO_2_, whereas the electron is promoted to the conduction band (CB) and the positive hole is made in the valence band. Then, excited electrons and holes will be recombined and disperse the input energy as heat. Then, electron and holes get trapped in metastable surface or react with electron donors and electron acceptors adsorbed on the photocatalyst surface. After reaction with water, these holes produce hydroxyl radicals that are important in the photocatalytic reaction. This reaction will continuously happen as long as the holes and electrons are present, so that it is important to have a long life of holes and electrons allowing them to migrate to the TiO_2_ particle surface and thus get trapped by the surface active sites. However, TiO_2_ is inactive due to faster electron-hole recombination than the charge transfer process and low adsorption ability thus reduces the photocatalytic reaction rate [5, 6]. On top of that, large band gap energy of TiO_2_ also leads to a low degradation rate because only a small percentage of solar spectrums in the UV region could be absorbed by TiO_2_.

Therefore, various strategies have been studied to improve the properties of bare TiO_2_, thus improved their photocatalytic efficiency as photocatalyst. All strategies are focused on reducing band gap, avoid fast electron-hole recombination, and increase the reactive sites on the surface of TiO_2_. Previously, researchers reported that Fe ion-doped TiO_2_/spherical activated carbon (SAC) exhibited high total organic carbon (TOC) and chemical oxygen demand (COD) removal on the photodegradation of humic acid under 315~400 nm wavelength irradiation because the Fe ion doping helps in reducing the band gap energy and enhancing the separation of photogenerated electron-hole charges [7], while graphene oxide combined with TiO_2_ (GO/TiO_2_) nanocomposite enhanced the oil recovery up to 65% of the original oil volume as compared to 56% for pure GO augment solution [8]. Besides that, a ZrO_2_-doped HZSM-5 catalyst shows high catalytic activity for methanol conversion and dimethyl ether production [9], suggesting that metal ion and metal oxide doping increased the performance of the materials. However, when the concentration of doping exceeds the optimum amount, the doping materials mainly act as a recombination center for generated electrons and holes, thus reduced the catalytic activity [10].

Recently, unique TiO_2_ nanostructures like nanoparticles [11], nanotubes [12], nanofibers [13], nanowires [14], and nanorods [15] have been synthesized using several methods including sol-gel, hydrothermal, spin coating, microwave-assisted solvothermal, and metal organic chemical vapor deposition methods. However, a hydrothermal method, in particular, showed high potential for morphology control of TiO_2_ and it is possible to grow different nanostructures by dissolution and crystallization of precursors, thereby creating distinctive difference in their characteristics at nanoscale level. Different nanostructured materials possessed different morphologies and characteristics that affected the performance of the synthesized nanostructure materials significantly. For these reasons, TiO_2_ nanostructures were used in various applications such as photocatalytic processes [16], photovoltaic [17], and sensor [18]. In general, TiO_2_ nanostructures can be categorized into zero- (0D), one- (1D), two- (2D), and three-dimensional (3D) materials. The materials with all the dimensions within the nanoscale (≤100 nm) size are classified as 0D nanostructure materials. 1D nanostructure materials are the materials which have one dimension of their structure that is outside the nanoscale. While 2D nanostructure materials are those in which two of the dimensions are not confined to the nanoscale. On the other hand, 3D materials are the materials constructed from these low dimensional materials.

1D and 2D nanostructures offer high rate of charge carrier transportation high interfacial free energies due to providing direct pathways for these photogenerated charge carrier movement [19, 20]. They also have high interfacial free energies, resulting in their aggregation and loss of active surface area during storage or photocatalytic testing [21]. To overcome this limitation, researchers are now actively exploring hierarchical photocatalyst structures, which contain 1D or 2D TiO_2_ components organized into more complex 3D architectures. 3D flower-like nanostructures, with 1D or 2D nanostructured components as “petals”, are promising in this regard. Thus, in this study, 3D flower-like anatase TiO_2_ nanostructures were synthesized, characterized, and tested as photocatalysts for methyl orange degradation and as antibacterial materials. Researchers reported that brookite TiO_2_ nanoflowers could be synthesized using TiOSO_4_ as a precursor material using a hydrothermal method and the product exhibits larger light adsorption and larger permittivity than anatase TiO_2_ powder [22, 23], while Liu and coworkers found out that the photochemical degradation rate of anatase flower-like TiO_2_ hierarchical structures on methylene blue is two times that of commercial P25 TiO_2_ powders [24]. They used Ti powder as a precursor to produce flower-like TiO_2_ with high specific surface area and a large light harvesting efficiency.

To the best of our knowledge, most studies were carried out to investigate the properties and application of nanoflower TiO_2_ as a photocatalyst, but no study has been conducted on antibacterial properties of the nanostructures. Therefore, this paper reported dual application of flower-like TiO_2_ nanostructures as photocatalytic and antibacterial materials due to their unique physical and chemical properties. In this study, titanium tetraisopropoxide has been used as a precursor for flower-like TiO_2_ nanostructure synthesis because it is easy to be hydrolysed to produce TiO_2_ particles, is inexpensive, and is nontoxic as compared to other precursors such as TiOSO_4_, Ti powder, and TiCl_4_.

## 2. Materials and Methods

### 2.1. Preparation of the TiO_2_ Photocatalyst

Titanium tetraisopropoxide, Ti(OC_3_H_7_)_4_, and distilled water were mixed with a molar ratio of 1 : 4, and the pH of mixture was adjusted using hydrochloric acid (HCl) of 0.2 molarity for restraining the hydrolysis process of the solution. The solution was stirred continuously at slow speed for 30 min and transferred into Teflon-lined autoclave. A 100 ml of HCl (0.5 M) was added and stirred for 15 to produce homogeneous solution in Teflon-lined autoclave. The acidic condition of solution (pH = ~2) is important in order to produce the flower-like morphology of TiO_2_ using a hydrothermal method. The Teflon-lined autoclave containing the solution was putted in the furnace for hydrothermal treatment at 150°C overnight. After the reaction, the product was washed with distilled water until pH 7 of washing solution was obtained. Then, the white solid was separated and collected from solution and subsequently dried at 80°C for 24 h. After drying, the obtained powder was calcined at 500°C in air for 2 hours for characterization.

### 2.2. Characterization

Raman analysis was carried out at room temperature using a Horiba Jobin-Yvon HR800 UV Raman spectrometer. The data was collected using an increment of 1 cm^−1^ and an integration time of 1 second. The wavelength was obtained from 100 to 1000 cm^−1^. Bruker D8 diffractometer with Cu-K*α* (*λ* = 1.54021 Å) X-ray powder diffraction (XRD) was used for a crystal structure study. Scans were performed in a step of 0.2°/second over the range of 2*θ* from 20 up to 80°. The morphology of the sample and images was captured by a ZEISS SUPRA™ 35VP field emission scanning electron microscope (FESEM). The energy-dispersive X-ray spectroscopy (EDX) for the elemental composition was analyzed using GENESIS 2000 X-ray Microanalysis System (XMS) which is equipped with SUPRA™ 35VP FESEM. High-resolution transmission electron microscopy (HRTEM) Philips CM12 was used for phase structure identification. The crystallographic structure of the samples was further investigated by selected area electron diffraction (SAED). Micromeritics ASAP 2000 instrument was used for the nitrogen gas adsorption analysis at the temperature of -196°C (boiling temperature of liquid nitrogen) in order to determine the Brunauer-Emmett-Teller (BET) surface area and porosity. A PerkinElmer Lambda 35 UV-Vis spectrometer was used for band gap measurements. The elemental and oxidation state analyses were conducted using X-ray photoelectron spectroscopy (XPS) Ultra Axis PLD, Kratos, employing Al (mono) K*α* radiation (BE = 1486.7 eV). The broad survey scan was performed in the energy range from 0 to 1000 eV. The binding energy (BE) for the samples was calibrated by setting the BE of C 1s to 284.6 eV.

### 2.3. Photocatalytic Study

The photocatalytic activity of the synthesized TiO_2_ and commercial TiO_2_ is evaluated for the photodegradation of methyl orange (MO) dye. For photodegradation, 10 mg powder of each catalyst was separately dispersed in a 60 ml MO dye aqueous solution at a specific concentration (5 ppm). Proceeding to the photocatalytic test, the mixture solution was stirred magnetically in dark for 1 h to attain adsorption/desorption equilibrium between dye and catalyst. Then, the solution was exposed to 8 W UV lamp as the source of irradiation for 1 h. A 5 ml sample was drawn at every 10 minutes interval for absorbance measurement. The removed suspensions were centrifuged (6000 rpm for 10 min) for solid-liquid separation. The clear solution was analyzed using UV-Vis spectrometer (PerkinElmer Lambda 35 UV-Vis) to quantify the extent of dye degradation. The photodegradation efficiency (*η*) was calculated using Equation (1):

(1) $$\begin{array}{c} \text{Degradation }\left(\eta \right)=\frac{C_{0}-C_{t}}{C_{0}}\times 100, \end{array}$$

where *C*_0_ is the initial absorption of MO and *C*_*t*_ is the absorption of MO after the reaction at *t* time.

### 2.4. Antibacterial Study

For antibacterial study, gram-positive Staphylococcus aureus microbes were used for an antibacterial assay. The standard growth medium (Mueller-Hinton, MH, Difco™) agar was prepared by sterilizing with an autoclave for 15 min at 120°C. Prior to the bacterial inoculation, the Staphylococcus aureus is subculture in MH agar and was incubated aerobically at 37°C for 24 h to make sure the bacteria are in a stable condition without any contamination. The bacterial concentrations were measured via simple optical density measurement using a BioMerieux DensiCHEK Plus spectrophotometer at 600 nm. In this study, the bacterial suspensions were adjusted to equivalent turbidity at 0.5 McFarland standards. Inoculants of Staphylococcus aureus were evenly spread in sterile petri plates contained in the MH agar. Using a sterile cotton swab, all bacteria were swabbed over the surface of the agar plates. 0.1 mg of flower-like TiO_2_ nanostructures, commercial TiO_2_ powder sample, and penicillin as the control were gently pressed on the agar. The penicillin was used as a standard antibiotic for the positive control in this study. The plates contained samples and agar with bacteria were incubated at 37°C for 24 h in triplicates. The observations on clear zone of each plate were recorded after 24 h incubation at 37°C. It is recorded as an indication of growth against microbial species.

## 3. Results and Discussion

**Figure 1:**
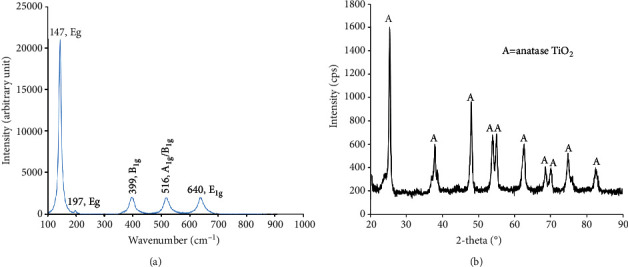
(a) Raman spectrum and (b) XRD pattern of flower-like TiO_2_ nanostructures.

Figure 1(a) shows the Raman spectra of the synthesized sample. The peaks at 147 cm^−1^ (*E*_*g*_), 197 cm^−1^ (*E*_*g*_), 399 cm^−1^ (*B*_1*g*_), 516 cm^−1^ (*A*_1*g*_/*B*_1*g*_ unresolved doublet), and 640 cm^−1^(*E*_*g*_) were observed which are assigned to TiO_2_ anatase which is in good agreement with the published data [25]. Furthermore, the strongest peak at 147 cm^−1^ (*E*_*g*_) was attributed to the external vibration of the anatase structure; therefore, it could be concluded that the prepared sample is an anatase TiO_2_ [26, 27]. This result was supported by XRD result as sharp and narrow peaks were appeared at 2*θ* of 25.57°, 38.05°, 48.28°, 54.10°, 55.29°, 62.90°, 68.92°, 70.45°, 75.24°, and 82.83° (Figure 1(b)), which were indexed to the diffraction planes (101), (112), (200), (105), (211), (204), (116), (220), and (215) assigned to tetragonal structure of anatase TiO_2_ (JCPDS # 02-0406), respectively. This finding proved that the synthesized sample is TiO_2_ anatase phase. It was well reported that anatase TiO_2_ displays better photocatalytic activity as compared to other phases of TiO_2_-like rutile and brookite due to their higher electron mobility [28]. Thus, formation of anatase TiO_2_ in this research could enhance the photocatalytic activity to degrade methylene blue and bacterial decomposition.

**Figure 2:**
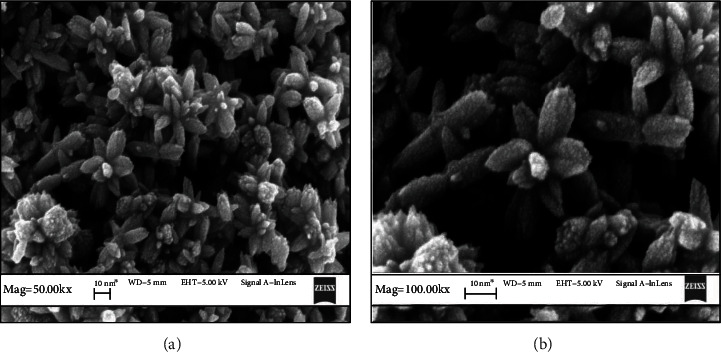
FESEM of flower-like anatase TiO_2_ nanostructures at (a) 50 K (lower magnification) and (b) 100 K (higher magnification).

**Figure 3:**
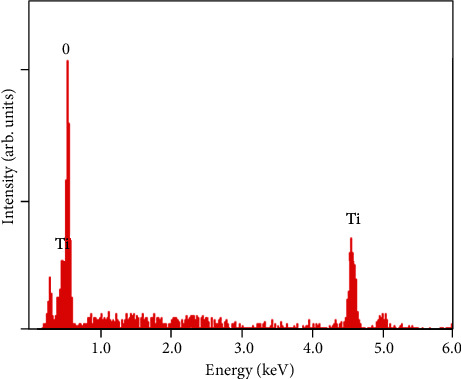
EDS spectrum of flower-like anatase TiO_2_ nanostructures.

The FESEM image of anatase TiO_2_ is depicted in Figure 2. Homogeneous flower-like TiO_2_ nanostructure was observed, and the diameter and length of flower-like TiO_2_ were found to be within 80-100 nm and 150–200 nm, respectively. Flower-like nanostructures are interlaced and stacked together, exhibiting a good dispersion. The image in Figure 2(a) was taken at lower magnification, while the other images (Figure 2(b) were captured at higher magnification. It can be seen in the SEM micrographs that the petals of the flowers were in the size range of 20–90 nm. The formation of flower-like nanostructures can be explained by multistep growth mechanism. First, under high-temperature hydrothermal conditions, the reaction system has crystal nuclei, because the system has a large surface energy at this time, the rapid growth of crystal nuclei into titanium dioxide particles. In order to reduce the surface energy of the system, the titanium dioxide particles are self-assembled together to form the ball aggregation structure. The titanium dioxide particles continue to produce, attached to the ball aggregate structure around it, and its structure begin to change; the morphology of product began to become ellipsoid, when the hydrothermal time is longer; the nucleation process of the product has been completed. In this process, the titanium dioxide particles simultaneously self-assembled to form nanorods adhered to the aggregate structure of the nanocrystal. And overtime flower-like TiO_2_ nanostructures were formed. The formation of flower-like TiO_2_ nanostructures is good for photocatalytic activity because it will help to maximize the light harvesting due to the multiple reflections of light within the interior nanoflowers [29]. The elemental analysis of the synthesized TiO_2_ nanoflowers was carried out using energy-dispersive X-ray spectroscopy (EDS). As can be seen in Figure 3, only oxygen and titanium elements were present, with no evidence of any significant impurities. Therefore, it was confirmed that the synthesized TiO_2_ nanoflower was pure titanium dioxide with no impurities.

**Figure 4:**
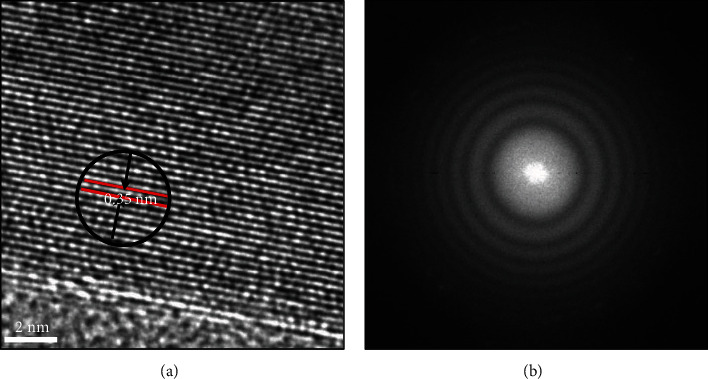
(a) HRTEM micrographs and (b) SAED pattern of flower-like TiO_2_ nanostructures.

The purity of the sample was investigated further using HRTEM. The lattice fringes of flower-like TiO_2_ nanostructures are clearly observed in Figure 4(a), which indicated that these samples had high degrees of crystallinity and phase purity. The lattice fringe distance (*d* spacing) was determined to be about 0.35 nm, which could be undoubtedly assigned to the lattice facet of anatase TiO_2_ hexagonal (1 0 1) [30], while Figure 4(b) illustrates the selected area electron diffraction (SAED) patterns of the sample. A series of rings were observed which confirmed the polycrystalline nature. The SAED pattern displays five fringe patterns with spacing of 3.512 Å, 2.293 Å, 1.841 Å, 1.626 Å, and 1.442 Å which were consistent with (0 1 1), (1 0 1), (2 0 0), (2 1 1), and (0 0 2) of anatase TiO_2_*h* *k* *l* index, respectively. The result of these investigations is consistent with the Raman and XRD results obtained.

**Figure 5:**
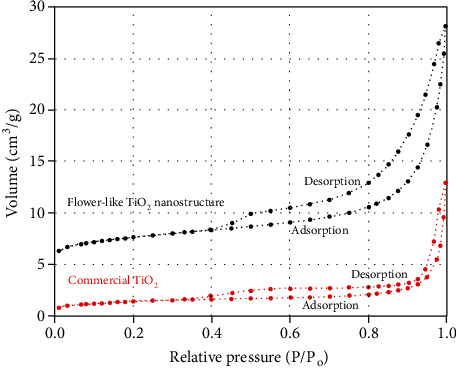
Isotherm plot of flower-like TiO_2_ nanostructure and commercial TiO_2_.

The BET surface areas of flower-like rutile TiO_2_ nanostructures and commercial TiO_2_ were 108.24 m^2^/g and 23.12 m^2^/g, respectively (Table 1). Higher surface area of the TiO_2_ nanostructure attributed to their flower-like morphological structure which is able to prevent the agglomeration of the particles. Similar trend was observed on pore diameter and pore volume of the studied samples. Larger pore volume of flower-like TiO_2_ nanostructures as compared to commercial TiO_2_ is due to their elongated morphology and smaller particles.  Figure 5 shows the isotherm plot of the samples, while types of isotherms, hysteresis, pores, and shape of pores of flower-like TiO_2_ nanostructure and commercial TiO_2_ are tabulated in Table 2. Both samples exhibit similar types of isotherms, having a type IV isotherm indicating the presence of mesopore in the system which supports the result of pore diameter of the samples. Mesopore materials have pore sizes generally in the range of 2 to 50 nm. For type IV isotherm, it could be observed that the adsorption of the nitrogen gas is low at the initial relative pressure (*P*/*P*_*o*_), but then increases markedly at higher values of *P*/*P*_*o*_ where pore capillary condensation takes place. A hysteresis effect associated with the pore condensation and studied samples displays closely with type H3 hysteresis loops, implying the slit-shaped mesoporous characteristics of materials [31].

**Figure 6:**
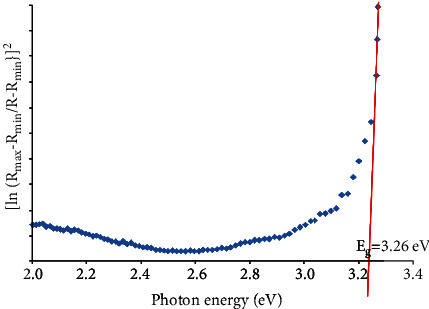
Band gap energy of flower-like TiO_2_ nanostructures.

As shown in Figure 6, the band energy of flower-like TiO_2_ nanostructures is 3.26 eV slightly larger than anatase TiO_2_ (3.2 eV) as reported by previous researchers [32]. Their large band gap energy is due to the size quantization in nanomaterials. In nanosized materials, the effect of size quantization was due to localization of electrons and positive holes in a confined volume of the materials. This will result in a change of energy band structure, due to the separation of individual energy levels and an increase in effective optical band gap of the nanomaterials as compared with bulk materials [33]. In general, the ideal band gap is less than 3.1 eV, because the materials with lower band gap could demonstrate the photocatalytic properties under ultraviolet (UV) and visible light as well as sunlight. Therefore, more efficient materials could be produced.

**Figure 7:**
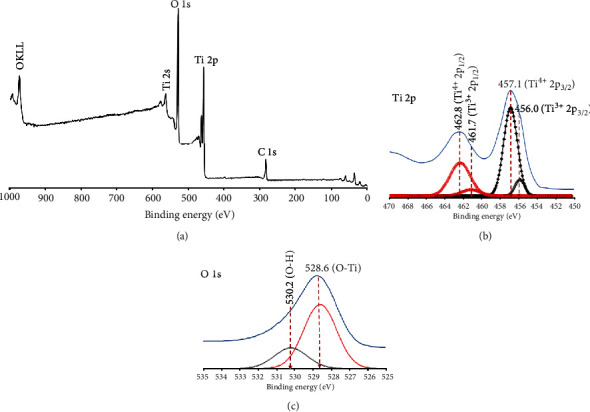
(a) XPS spectrum and (b, c) high-resolution XPS of flower-like anatase TiO_2_ nanostructure.

The XPS survey spectrum of flower-like TiO_2_ nanostructures is shown in Figure 7(a). The peak appearances which are attributed to Ti 2p, O 1s, and C 1s reveal the existence of Ti, O, and C on the surface of the sample. Ti and O elements correspond to the synthesized sample, while the presence of C element was ascribed to adventitious carbon from the XPS [34].  Figure 7(b) shows the high-resolution XPS spectra of Ti 2p. An unsymmetrical peak was observed, which means that there exist two chemical forms of Ti in the sample. Two strong peaks located at 457.1 and 462.8 eV binding energies were attributed to the Ti^4+^ 2p_1/2_ and Ti^4+^ 2p_3/2_ spin orbital splitting photoelectrons, respectively, showing the presence of Ti^4+^ [35]. Besides these predominant Ti^4+^ peaks, a minor species of titanium was also found located at 456.0 and 461.7 eV binding energies. These could be identified as Ti^3+^ 2p_3/2_ and Ti^3+^ 2p_1/2_, respectively, suggesting the presence of Ti^3+^ species in the sample [36]. Interestingly, the existence of Ti^3+^ surface state can act as electron trapper to prevent the recombination of electron (e^−^) and positive holes (h^+^) [37]. As shown in Figure 7(c), after deconvolution of high-resolution XPS spectra O 1s, two peaks were observed. The main peak of O 1s is located at about 528.6 eV, which corresponds to lattice oxygen of TiO_2_ [O–Ti], while another one shoulder peak at higher binding energy (530.2 eV) could be identified attributed to the hydroxyl surface species of [O–H] [34]. The presence of the hydroxyl groups can enhance the performance of the photocatalyst due to the longer electron lifetime [38].

**Figure 8:**
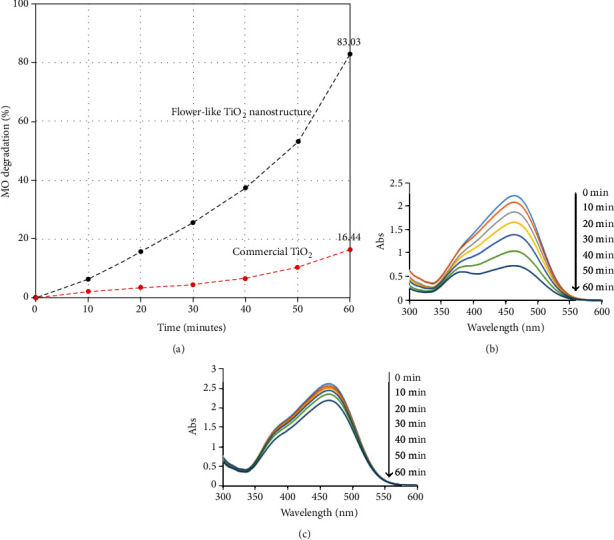
(a) Photocatalytic degradation of MO, (b) UV-Vis spectra of MO degradation by flower-like TiO_2_ nanostructures, and (c) UV-Vis spectra of MO degradation by commercial TiO_2_.

For photocatalytic activity study, MO was used as the targeted pollutant because MO was widely used in various industries such as textile and food processing for coloring. Due to their high stability against photolysis and self-degradation, it is remained in an aqueous solution for a long time. This situation will contribute to the water pollution and toxicity to aquatic life. Therefore, the MO is an appropriate pollutant to evaluate the photocatalytic activity of materials.  Figure 8(a) shows the photocatalytic degradation rate of methyl orange (MO) under ultraviolet (UV) light using various photocatalysts. After 60 minutes, 83.03% and 16.44% of MO were degraded using flower-like TiO_2_ nanostructure and commercial TiO_2_, respectively. Their UV-Vis spectra of MO degradation are shown in Figures 8(b) and 8(c), respectively. The degradation of MO did not reach the stable trend because the degradation still can be increased if the reaction was carried out over a longer period suggesting the performance of the photocatalyst remained during the reaction. For comparison, the degradation rate of MO using other TiO_2_ nanostructures is listed in Table 3. The flower-like TiO_2_ nanostructure exhibits better photocatalytic activity in MO degradation.

The result shows that the flower-like TiO_2_ nanostructure could act as an effective photocatalyst to degrade MO. An ordered and strongly interconnected nanoflower architecture offers the potential for improved electron transport leading to higher degradation efficiencies. The flower-like TiO_2_ nanostructure also composed of the very fine rice-like nanorods; consequently, photoinduced carriers are easier to transfer to the surface and initiate the reactions, thus reducing the recombination of photogenerated electrons and holes [43, 44]. Moreover, the layered structure of flower-like TiO_2_ can provide sufficient space to polarize the related atoms and orbitals, which can effectively separate the photogenerated electron-hole pairs, thereby enhanced the photocatalytic activity [45]. On the other hand, low photocatalytic activity of commercial TiO_2_ is due to their low surface area and zero dimensional (0D) materials (spherical shape of TiO_2_). It is well known that the structural disorder at the contact between two crystalline TiO_2_ nanoparticles leads to enhanced scattering of free electrons, thus reducing electron mobility. Moreover, the short charge-separation distances within the particle as well as the rapid charge-recombination speed led to a low quantum yield, thus reduces the photocatalytic activity of TiO_2_ particles [46].

**Figure 9:**
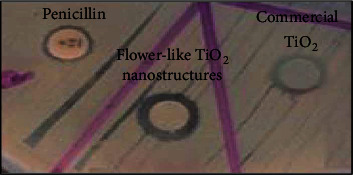
Agar well diffusion test results of penicillin, flower-like TiO_2_ nanostructure, and commercial TiO_2_ against Staphylococcus aureus.

The antibacterial activity of flower-like TiO_2_ nanostructure and commercial TiO_2_ was studied against gram-positive Staphylococcus aureus by a qualitative disk method, and their activity was compared with control sample (penicillin) (Figure 9). The inhibition zone was found to be 10 ± 0.02 mm, 13 ± 0.02 mm, and 11 ± 0.06 mm after 24 hours for penicillin, flower-like TiO_2_ nanostructure, and commercial TiO_2_, respectively, as tabulated in Table 4. The result obtained suggests that the synthesized flower-like TiO_2_ nanostructure and commercial TiO_2_ were better than penicillin as antibacterial materials. This is due to the capability of TiO_2_ in dissolving the outer membranes of bacteria by using reactive oxygen species (ROS) [47]. It is well known that TiO_2_ material is a very good oxidation agent to produce ROS by reacting with water (H_2_O) and oxygen (O_2_). ROS (·OH, H_2_O_2_, and ·O_2_-) are formed when these photogenerated charge carriers (e^−^_CB_ in conduction band and h_VB_ in valence band) interact with water and dissolved O_2_ molecules. These ROS interact with the cell wall and cell membrane of bacteria and damage them. ROS oxidizes the membrane phosphatidylethanolamine to form malondialdehyde which is a lipid peroxidation product [48]. This malondialdehyde acts as an index to measure the peroxidation and cell damage. Electrostatic force of interaction between the bacteria and photocatalyst is important for enhanced antibacterial activity, due to which catalyst penetrates inside the cell causing more damage to the membrane. This damage to the cell wall and cell membrane leads to the leakage of intracellular substances (K^+^). This K^+^ is the universal substance in bacteria which helps in protein synthesis and regulation of polysome content. Further, cell components like genomic material (DNA and RNA), proteins, and ribosomes are also damaged by these ROS due to altered cell permeability [49]. Flower-like TiO_2_ nanostructure shows the highest antibacterial activity because of their larger surface area which helps generating large number of ROS and consequently exhibits better antibacterial activity. On top of that, a flower-like TiO_2_ superstructure, or, in other words, a 3D micro-nanomaterial with a hierarchical structure, had good light absorption efficiency and appropriate refractive index to generate more photocarries for ROS production.

## 4. Conclusions

Flower-like TiO_2_ nanostructures with anatase phase were synthesized via a hydrothermal method using titanium isopropoxide as precursors. The existence of anatase TiO_2_ was proved by Raman and XRD results as sharp and narrow peaks appeared assigned to anatase TiO_2_. FESEM images have shown the existence of flower-like TiO_2_ nanostructures with 80-90 nm in diameter and 150-200 nm in length. The BET surface area and pore volume of the nanostructures were found to be 108.4 m^2^g^−1^ and 0.688 m^3^g^−1^, respectively. High surface area, pore volume, and anatase phase of flower-like TiO_2_ nanostructure support their good performance for MO degradation (83.03%) in 60 minutes and as antibacterial material with 13.00 ± 0.06 mm inhibition zone against Staphylococcus aureus. The presence of Ti^4+^ and Ti^3+^ in the flower-like TiO_2_ nanostructures as confirmed by XPS analysis could retard the charge recombination and improve the photogenerated electrons to increase the generation of reactive oxygen species (ROS) for degradation of MO and bacteria.

## Figures and Tables

**Table 1:** Nitrogen adsorption analysis of flower-like TiO_2_ nanostructure and commercial TiO_2_.

Sample	BET surface area (m^2^/g)	Pore volume (cm^3^/g)	Pore diameter (nm)
Commercial TiO_2_	23.12	0.102	4.224
Flower-like TiO_2_ nanostructure	108.24	0.688	6.228

**Table 2:** Types of isotherms, hysteresis, pores, and shape of pores of flower-like TiO_2_ nanostructure and commercial TiO_2_.

Sample	Type of isotherms	Type of hysteresis	Type of pores	Shape of pores
Commercial TiO_2_	IV	H3	Mesopore	Slit-shaped pores
Flower-like TiO_2_ nanostructure	IV	H3	Mesopore	Slit-shaped pores

**Table 3:** Degradation of MO using flower-like TiO_2_ and other nanostructure photocatalysts.

Photocatalyst	MO degradation (%)	Reference
Flower-like TiO_2_ nanostructures	83.03 after 60 minutes	This study
TiO_2_ nanorods	51 after 150 minutes	[39]
TiO_2_ nanowires	40 after 120 minutes	[40]
N-doped TiO_2_ nanowires	~55 after 60 minutes	[41]
Mesoporous TiO_2_/MoS/TiO_2_ nanosheet	~60 after 60 minutes	[42]

**Table 4:** Inhibition zone of penicillin, flower-like TiO_2_ nanostructure, and commercial TiO_2_ against Staphylococcus aureus.

Sample	Penicillin	Flower-like TiO_2_	Commercial TiO_2_
Inhibition zone (mm)	10.00 ± 0.02	13.00 ± 0.02	11.00 ± 0.06

## Data Availability

The data has been included in the manuscript.
